# LIM-kinase 1 effects on memory abilities and male courtship song in Drosophila depend on the neuronal type

**DOI:** 10.18699/VJGB-23-31

**Published:** 2023-06

**Authors:** A.V. Zhuravlev, E.S. Zalomaeva, E.S. Egozova, V.V. Sokurova, E.A. Nikitina, E.V. Savvateeva-Popova

**Affiliations:** Pavlov Institute of Physiology of the Russian Academy of Sciences, St. Petersburg, Russia; Pavlov Institute of Physiology of the Russian Academy of Sciences, St. Petersburg, RussiaHerzen State Pedagogical University of Russia, St. Petersburg, Russia; Herzen State Pedagogical University of Russia, St. Petersburg, Russia; Herzen State Pedagogical University of Russia, St. Petersburg, Russia; Pavlov Institute of Physiology of the Russian Academy of Sciences, St. Petersburg, Russia Herzen State Pedagogical University of Russia, St. Petersburg, Russia; Pavlov Institute of Physiology of the Russian Academy of Sciences, St. Petersburg, Russia

**Keywords:** Drosophila, LIMK1, conditioned courtship suppression paradigm, memory, forgetting, dopaminergic neurons, cholinergic neurons, fruitless, male courtship song, дрозофила, LIMK1, условно-рефлекторное подавление ухаживания, память, забывание, дофаминергические нейроны, холинергические нейроны, fruitless, брачная песня самца

## Abstract

The signal pathway of actin remodeling, including LIM-kinase 1 (LIMK1) and its substrate cofilin, regulates multiple processes in neurons of vertebrates and invertebrates. Drosophila melanogaster is widely used as a model object for studying mechanisms of memory formation, storage, retrieval and forgetting. Previously, active forgetting in Drosophila was investigated in the standard Pavlovian olfactory conditioning paradigm. The role of specific dopaminergic neurons (DAN) and components of the actin remodeling pathway in different forms of forgetting was shown. In our research, we investigated the role of LIMK1 in Drosophila memory and forgetting in the conditioned courtship suppression paradigm (CCSP). In the Drosophila brain, LIMK1 and p-cofilin levels appeared to be low in specific neuropil structures, including the mushroom body (MB) lobes and the central complex. At the same time, LIMK1 was observed in cell bodies, such as DAN clusters regulating memory formation in CCSP. We applied GAL4 × UAS binary system to induce limk1 RNA interference in different types of neurons. The hybrid strain with limk1 interference in MB lobes and glia showed an increase in 3-h short-term memory (STM), without significant effects on long-term memory. limk1 interference in cholinergic neurons (CHN) impaired STM, while its interference in DAN and serotoninergic neurons (SRN) also dramatically impaired the flies’ learning ability. By contrast, limk1 interference in fruitless neurons (FRN) resulted in increased 15–60 min STM, indicating a possible LIMK1 role in active forgetting. Males with limk1 interference in CHN and FRN also showed the opposite trends of courtship song parameters changes. Thus, LIMK1 effects on the Drosophila male memory and courtship song appeared to depend on the neuronal type or brain structure.

## Abstract (ru)

Сигнальный каскад ремоделирования актина, в состав которого входят LIM-киназа 1 (LIMK1) и ее субстрат кофилин, участвует в регуляции различных процессов в нейронах позвоночных и беспозвоночных животных. Drosophila melanogaster широко используется как модельный объект для изучения механизмов формирования, сохранения и воспроизведения памяти, а также забывания. Ранее активное забывание у дрозофилы исследовали с помощью классического павловского ольфакторного обучения. Было показано, что в разных формах забывания участвуют специфические дофаминергические нейроны и компоненты актинового каскада. В данной работе мы оценивали роль LIMK1 в процессах памяти и забывания у дрозофилы в парадигме условно-рефлекторного подавления ухаживания. В мозге дрозофилы уровень LIMK1 и фосфокофилина избирательно снижен в отдельных структурах нейропиля, включая лопасти грибовидных тел и центральный комплекс. В то же время LIMK1 присутствует в телах нервных клеток, таких как кластеры дофаминергических нейронов, регулирующие формирование памяти при условно-рефлекторном подавлении ухаживания. С использованием системы бинарного скрещивания GAL4 × UAS мы инициировали РНК-интерференцию limk1 в различных типах нервных клеток. У гибридных линий с интерференцией limk1 в лопастях грибовидных тел и глии наблюдалось усиление 3-часовой краткосрочной памяти, без видимого влияния на долгосрочную память. Интерференция limk1 в холинергических нейронах приводила к снижению краткосрочной памяти, в дофаминергических и серотонинергических нейронах ее результатом было также существенное нарушение способности мух к обучению. Напротив, интерференция limk1 в нейронах fruitless усиливала 15–60-минутную краткосрочную память, что указывает на возможную роль LIMK1 в процессах активного забывания. У самцов с интерференцией limk1 в холинергических и fruitless нейронах также были отмечены разнонаправленные изменения параметров брач- ной песни. Таким образом, эффекты LIMK1 на память и брачную песню самцов дрозофилы определяются типом нервных клеток или структурой мозга.

## Introduction

Memory formation and forgetting serve as the basis of behavioral plasticity. Whereas memory is a specific process of information acquisition, storage and retrieval by the nervous system, active forgetting is defined as “a mechanism or series of mechanisms to remove memories that become unused” (Davis, Zhong, 2017). Associative memory formation and active forgetting occur in both mammals and invertebrates, including Drosophila melanogaster (Medina, 2018), which is a well-known object of classical genetics. Having a short life cycle and relatively simple nervous system, the fruit fly makes it easy to perform genetic analysis of the molecular basis of behavioral and cognitive processes.

There are several experimental techniques to form associative memory in Drosophila, including short-term memory (STM) and protein synthesis-dependent long-term me-mory (LTM). The most widely used technique is classical Pavlovian learning with negative electroshock reinforcement, or olfactory aversive learning (OAVL), which revealed genes responsible for different types of memory (Tully et al., 1994). More natural is conditioned courtship suppression paradigm (CCSP) (Siegel, Hall, 1979; Kamyshev et al., 1999). GAL4 × UAS binary expression system (Duffy, 2002) is used to study the effects of specific genes on memory processes. The fine neural organization of the mushroom bodies (MB), a principle structure responsible for associative olfactory learning in Drosophila, was evaluated in detail. The MB output neurons (MBON) are the main effectors of MB, whereas specific clusters of dopaminergic neurons (DAN) regulate the activity of MB – MBON synaptic contacts (Aso et al., 2009, 2014a, b). Among them are aSP13 DAN of the protocerebral anterior medial cluster (PAM), which innervate γ5 area of MB, playing a crucial role in CCSP learning and memory (Keleman et al., 2012).

The molecular and neural mechanisms of active forgetting implicate the activity of DAN and Rac1-dependent signal pathways (Medina, 2018). Small GTPases of the Rho family, including Rho and Rac, regulate neuronal actin polymerization during the Drosophila nervous system development. Rho via its effector ROCK or Rac/Cdc42 via its effector Pak activate LIM-kinase 1 (LIMK1), which phosphorylates Drosophila cofilin (twinstar) protein, blocking its actin-depolymerization activity and inhibiting axon growth. Rac also acts through Pakindependent pathway to antagonize LIMK1 and promote axon growth (Ng, Luo, 2004). In addition to its role in neurogenesis, Rac is crucial for both interference-induced and passive forgetting in OAVL paradigm. PAK/LIMK1/cofilin pathway probably acts downstream Rac1 (Shuai et al., 2010). Forgetting specific types of memory depends on different signal proteins (Zhang et al., 2016; Gao et al., 2019).

Forgetting in OAVL paradigm is caused by several DAN of the protocerebral posterior lateral 1 (PPL1) cluster, which innervates some MB structures, such as pedunculus, lower and upper stalk. Memory acquisition and forgetting are regulated by different dopamine receptors, dDA1 and DAMB respectively (Berry et al., 2012). Coincidence of conditioned and unconditioned stimuli creates a memory trace in MBON- γ2αʹ1, probably inhibiting the MB > MBON-γ2αʹ1 synapses. The unconditioned stimulus alone activates DAN-γ2αʹ1, which in turn disinhibit MB > MBON-γ2αʹ1 synapses and cause forgetting (Berry et al., 2018). DAN that innervate the MB ααʹ tip induce the interference-based forgetting through the scaffold protein Scribble, binding together Rac1, PAK3 and cofilin (Cervantes-Sandoval et al., 2016).

Whereas multiple data prove the importance of DAN and actin-remodeling signal pathway for forgetting in OAVL paradigm, there is virtually no data for molecular mechanisms of memory decay in CCSP. Effects of LIMK1-dependent signal cascade on CCSP learning and memory were firstly shown for the temperature-sensitive mutant agn ts3, with LIMK1 increase in the adult brain compared to the wild type Canton-S (CS). Temperature rise leads to a decrease in agn ts3 LIMK1 level, simultaneously restoring its learning ability and 3 h memory, which are drastically impaired in the norm (Medvedeva et al., 2008). agn ts3 has multiple polymorphisms within and near limk1 gene, as well as a changed profile of microRNA expression, and can serve as a model object for Williams syndrome (Nikitina et al., 2014; Savvateeva-Popova et al., 2017). The temporal profile of STM learning index (LI) was assayed in CCSP for agn ts3, as well as for the wild-type strains with limk1 polymorphisms, CS and Oregon R. Only CS was able to learn and store memory up to 24 h (Zalomaeva et al., 2021).

The behavioral effects of LIMK1 changes in agn ts3 do not give information about specific cell types, where LIMK1 can be involved in learning and memory. In this study, we performed the analysis of memory decay for several Drosophila GAL4 × UAS strains with neuronal type-specific limk1 RNA interference. LIMK1 distribution in the Drosophila brain structures was studied in detail using confocal microscopy. The effect of limk1 interference on fly memory ability depended on both neural type and memory form. LIMK1 also appeared to be involved in regulation of male courtship song: limk1 interference in different neuronal types specifically affected some song parameters.

## Materials and methods

Drosophila strains. Fly strains were provided by the Research Center “Biocollection of Pavlov Institute of Physiology RAS for the study of integrative mechanisms of the nervous and visceral systems”. The strain numbers (#) are given in accordance with the Research Center and Bloomington Drosophila Stock Center, USA (Cook et al., 2010). The following strains were used:

1. Сanton-S (СS) – the wild-type strain with limk1 polymorphisms.

2. agn ts3 – temperature-sensitive mutation on CS genetic background with limk1 polymorphisms, characterized by learning and memory defects.

3. Strains expressing GAL4 in specific neuronal types: #6794: w[*]; P{w[+mC]=nrv2-GAL4.S}8 P{w[+mC]= UAS-GFP.S65T}eg[T10]. GAL4 and green fluorescent protein (GFP) are expressed in nervous system under Nrv2 regulatory element; #6793: w[*]; P{w[+mC]=ChAT-GAL4.7.4}19B P{w[+mC]=UAS-GFP.S65T}Myo31DF[T2]. GAL4 and GFP are expressed in cholinergic neurons (CHN) under ChAT and VAChT regulatory elements; #7009: w[1118]; P{w[+mC]=Ddc-GAL4.L}Lmpt[4.36]. GAL4 is expressed in dopaminergic (DAN) and serotoninergic (SRN) neurons; #30027: w[1118]; P{w[+mW.hs]=GawB}fru[NP0021]. GAL4 is expressed in fruitless neurons regulating mating behavior.

4. Act-GAL4: w[1118]; P{w[+mC]=} 25FO1/CyO, y[+]; Canton-S background. GAL4 is expressed in the whole body under actin promoter.

5. Strains with UAS-dependent limk1 suppression: #26294: y[1] v[1]; P{y[+t7.7] v[+t1.8]=TRiP.JF02063} attP2. The strain expresses interfering RNA against limk1 (RNAi) under UAS (limk1-KD, knockdown). #36303: y[1] v[1]; P{y[+t7.7]=CaryP}attP2. The control strain with genetic background identic to that for #26294, but lacking RNAi (limk1“+”).

6. Strains with GFP gene regulated by UAS: #32186: w[*]; P{y[+t7.7] w[+mC]=10XUAS-IVS-mCD8:: GFP}attP40. #32202: w[*]; P{y[+t7.7] w[+mC]=10XUAS-IVS-GFPWPRE} attP2.

To induce limk1 RNA interference in specific neuronal types, a strain carrying GAL4 activator expressed under tissue- specific promoter was crossed to UAS strain #26294. The сross product of a GAL4 strain and #36303 strain served as a control.

Flies were raised on the standard yeast–raisin medium at 25 ± 0.5 °C and a 12:12 daily illumination cycle. For behavioral tests, experimental males were collected without anesthesia and kept individually. 5–6-day-old males were used in experiments. Females (CS) were collected as virgins and brought together with CS males for fertilization in CCSP one day before experiment.

Antibodies. Primary antibodies: Rat anti-LIMK1 multi-specific monoclonal (Enzo Life Sciences, ALX-803-343- C100); mouse anti-Drosophila cysteine string protein (CSP); rabbit anti-Drosophila tyrosine hydroxylase (TH) (Abcam, ab128249); rabbit anti-GFP (Abcam, ab290).

Secondary antibodies: Goat anti-mouse Alexa Fluor 488 (Invitrogen, A32723), donkey-anti-rat Alexa Fluor 594 (ThermoFisherScientific, A-21209), goat anti-rabbit Alexa Fluor 633 (Invitrogen, A21071).

RNA extraction and RT-PCR analysis of limk1 expression. The level of limk1 expression was assayed using semi-quantitative PCR in complex with reverse transcription (RT-PCR). Flies were anesthetized by freezing. 10 male flies or 70 male heads were homogenized in 300 μl TRI reagent (MRC, TR 118). Total RNA was extracted from homogenates according to the manufacturer’s protocol. The quality of RNA was checked by 1.5 % agarose gel electrophoresis. 1 μg RNA was reverse-transcribed by MMLV reverse transcriptase (Evrogen, #SK022S) according to the manufacturers’ protocol, using random hexamer primers and RNAse inhibitor (Syntol, #E-055). Semi-quantitative PCR was performed on a StepOne Plus (Applied Biosystems, Inc., USA) using qPCRmix HS SYBR+LowROX (Еvrogen, # PK156L) containing direct and reverse primers (0.5 mM each). Baseline and cycle threshold values were determined by automatic analysis using StepOne software v2.3 (Applied Biosystems, USA). rpl32 transcript was used as an internal control. The predesigned limk1 primers (PP12636 in FlyPrimerBank, http://www.flyrnai.org/ flyprimerbank) were used to bind all five limk1 cDNA isoforms, both premature and mature forms, as primers do not span the exon-intron borders. The relative limk1 transcript level was calculated using the comparative ΔΔCt method. The number of biological replicates (independent RNA extractions with reverse transcription) was 3–5, the number of technical replicates was 3.

The primer sequences were the following: rpl32: Forward: 5′-TATGCTAAGCTGTCGCACAAATGGC-3′ Reverse: 5′-GTTCTGCATGAGCAGGACCTCCA-3′ limk1: Forward: 5′-GTGAACGGCACACCAGTTAGT-3′ Reverse: 5′-ACTTGCACCGGATCATGCTC-3′ PCR parameters: 1. 1 cycle: 95 °С – 5 min. 2. 45 cycles. 95 °С – 20 s, 60 °С – 20 s, 72 °С – 20 s, 77 °С – 15 s (detection). 3. Melting curve: 95 °С – 15 s, 60 °С – 1 min, 60–95 °С (Δ 0.3 °С, 15 s).

Immunofluorescent staining of Drosophila brains. 5–6-day-old imago males were anesthetized by freezing. The brains were prepared in PBS buffer (pH 7.5) using needlesharp tweezers (Merck, T4412), fixed in 4 % paraformaldehyde in PBS for 1 h at RT and stained according to (Thapa et al., 2019), without a freezing stage. Antibodies were diluted in PBT (0.2 % Tween 20, 5 % BSA in PBS) as 1:200, for anti-CSP – 1:20. Previously, for better staining of brains, we increased the time of incubation with primary antibodies up to 5 days (Zhuravlev et al., 2020). Here, the incubation was performed at 4 °С for 3 days (with primary antibodies) or overnight (with secondary antibodies). Brains were mounted with Vectashield mounting medium containing DAPI (Vector laboratories, H-1200-10).

Protein distribution analysis in the brain by confocal microscopy. Brains were scanned frontally using laser scanning confocal microscopy (LSM 710 Carl Zeiss; Confocal microscopy Resource Center; Pavlov Institute of Physiology of the Russian Academy of Sciences, St. Petersburg, Russia). Scanning was performed using X63 objective at different depths (z-step 2 μm). Images were analyzed using Fiji software. The brain structures were visually mapped using the Drosophila brain online atlas (Virtual Fly Brain). To measure the average level of LIMK1 inside the brain structures, the average signal intensity was measured in three small square areas (~10 × 10 μm) within each of the structures. The average values were obtained and normalized to the average structure intensity for the given brain. Colocalization Threshold analysis was performed to measure co-localization of LIMK1 with neurospecific markers. To prepare figures, auto contrast function was used for each optical slice.

Learning and courtship suppression tests in Drosophila males. Flies learning and STM were estimated in CCSP, as described in (Zhuravlev et al., 2022). In the case of long term memory (LTM), learning was performed by placing flies in food-containing glasses (20 mm diam., ~20 mm high) for 5 h (Kamyshev et al., 1999). Courtship index (CI) and learning index (LI) were estimated at the following time points after learning: for short-term memory (STM) analysis: 0 min (learning), 3 h; for STM decay analysis: 15, 30, 60 min, 24 h; for LTM analysis: 0 min, 2 days, 8 days. In all groups, naive males (without mating experience) served as a control to calculate LI:

**Formula. 1.**
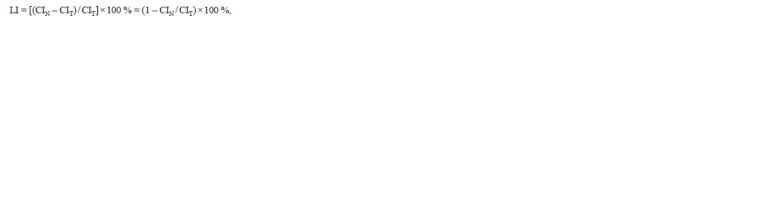
Formula1

where CIN is the middle CI for naive males, and CIT is the middle CI for males after training. The naive and trained males were the same age. The decrease in LI compared to LI (0 min) was considered a time-dependent memory decay. The decrease in LI for a mutant strain compared to that for the wild-type strain CS was considered a strain-specific impairment of learning or memory.

Courtship song analysis. The 5-day-old imago male courtship song was recorded as in (Savvateeva-Popova et al., 2008). A naive male of the studied line and a fertilized female (CS) were placed together in a Perplex chamber with a latticed bottom on top of a microphone. The chamber was placed in a foam box in a soundproof room. The sounds were recorded for 5 min using Audacity software (Mazzoni, Dannenberg, 2020). The sound signals were filtered to exclude noises, obtaining signals within 100–800 Hz. The level of noise was decreased using a standard Audacity plugin. The software Drosophila courtship song analysis (DCSA) (Iliadi et al., 2009) was used to automatically detect pulse and sine song components.

The results of analysis were manually edited. The mean values of the song parameters were calculated for each fly. The following parameters were estimated: pulse song index (PInd, % of the total time), pulse song initiation frequency (PFr; 100/s), sine song index (SInd, % of the total time), sine song frequency (SFr, 100/s), interpulse interval (IPI, ms), period of song pulse train (Per, s), intertrain interval (ITI, ms), train duration (TrainDur), pulse number in train (PulseN), sine song duration (SDur, ms), sine song amplitude (SAmp, C.U.), IPI variance (Var(IPI), ms2). Per is the time between the starts of the neighboring trains. ITI is the time between the end of the previous and the start of the next train.

Statistical analysis. Analysis of LIMK1 mRNA level was performed using two-sided t-test, Social Science Statistic online resource ( p < 0.05). Analysis of LI and courtship song parameters was performed using two-sided randomization test at significance level α of 0.05 (n = 20), using Drosophila Courtship Lite software (Nikolai Kamyshev, 2006, nkamster@ gmail.com), with 10000 iterations. The program is freely available from the author upon request. Randomization test was reported to be better for LI comparison than t-test or some nonparametric tests (Kamyshev et al., 1999). Courtship song parameters were also analyzed using two-sided Mann–Whitney U-test. Python 3 scripts were used to draw the box plots charts.

## Results

limk1 RNA level in Drosophila UAS × GAL4 hybrids

**Fig. 1.**
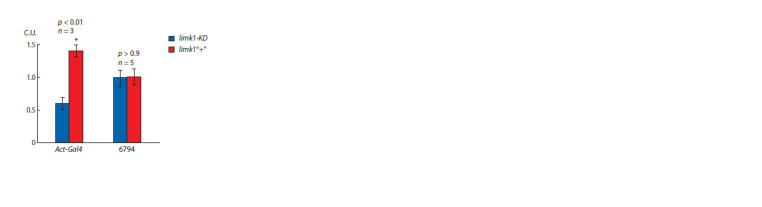
Comparative levels of limk1 RNA in the UAS > GAL4 hybrids with and without limk1 RNA interference X axis: GAL4 strains. Y axis, conditional units (C.U., expression levels are normalized to the average value). Statistical differences: * between limk1-KD > GAL4 and limk1“+” > GAL4 (two-sided t-test; p and n are shown above the charts). Standard error is shown.

To check that GAL4 really induces limk1 RNA interference in 26294 strain, we compared limk1 RNA level in the UAS (f) > GAL4 (m) hybrids. Females with and without transgenic RNAi for limk1 suppression (limk1-KD and limk1“+”, respectively) were crossed to Act-GAL4 males, expressing GAL4 in the whole body. The level of total limk1 RNA was approximately 2-fold lower in the hybrid with limk1 interference. These data confirmed the efficiency of RNAi-dependent limk1 suppression in 26294 strain (limk1-KD) upon its activation by GAL4. At the same time, there were no differences for limk1-KD > 6794 and limk1“+” > 6794, where RNA expression was measured in heads and was regulated by neuronal type-specific GAL4 (Fig. 1). Thus, limk1 RNA differences after neural type-specific limk1 RNA interference might be local or too low to be detected in the whole Drosophila heads.

LIMK1 distribution in the Drosophila brain

When studying LIMK1 distribution, we focused on the central part of the Drosophila brain, without the optic lobes (OL), mainly at the level of the superior medial protocerebrum (SMP) and gamma-lobes (γL) of MB. Here the PAM clusters of DAN are located (Mao, Davis, 2009), responsible for the Drosophila courtship learning and memory (Keleman et al., 2012). Additionally, the area including the central complex (CC) and calyx (Cal) surrounded by Kenyon cells (KC) was studied. The CSP-positive neuropil structures and tyrosine hydroxylase (TH)-positive DAN cell bodies and processes served as landmarks in describing the LIMK1 distribution. The following description is given for the wild-type strain CS (see the Table and Suppl. Material 1)1.

**Table 1.**
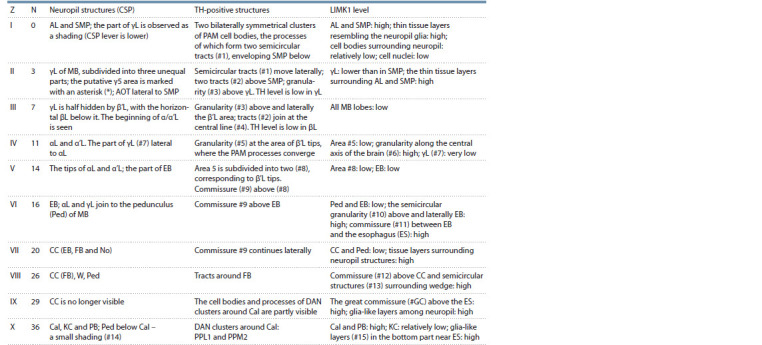
LIMK1 and TH distribution in the Canton-S brain (visual analysis) Note. The depth of the studied zone (Z) is given for the brain optical slices, from the PAM cell bodies to CC (I–V) or from CC to Cal (VI–X). N is the number of the brain optic slice for a given zone (step 2 μm). For different brains, there may be slight differences in depth of the given N. The brain structures (here and below): α, α’, β, β’ and γL – the corresponding lobes of MB, AL – the antennal lobes, AOT – the anterior optic tubercule, Cal – the calyx of MB, CC – the central complex, EB – the ellipsoid body of CC, ES – the esophagus, FB – the fan-shaped body of CC, KC – the Kenyon cells, MB – the mushroom body, MBl – the median bundle, No – the noduli of CC, PB – the protocerebral bridge, Ped – the pedunculus of MB, SEG – the subesophageal ganglion, SMP – the superior medial protocerebrum, W – wedge. DAN clusters, according to (Mao, Davis, 2009): PAM – the protocerebral anterior medial cluster, PPL – the protocerebral posterior lateral clusters, PPM – the protocerebral posterior medial clusters.

Supplementary Materials are available in the online version of the paper: https://vavilovj-icg.ru/download/pict-2023-27/appx9.pdf


**Fig. 2.**
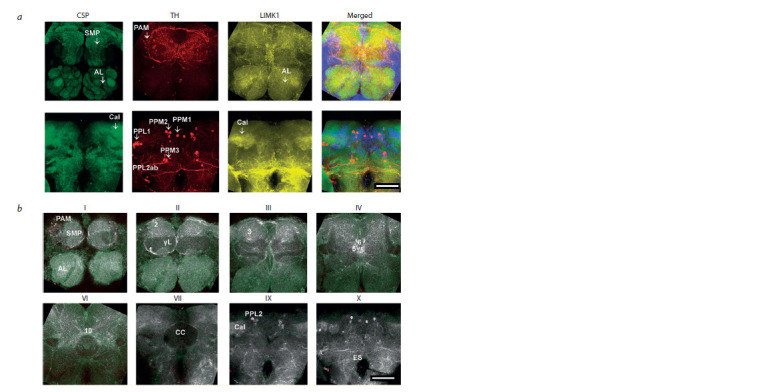
LIMK1 spatial distribution in the CS brain a, Z-projection. AL level (above) and Cal level (below). Color scheme: green – CSP, red – TH, yellow – LIMK1, blue – DAPI. b, Co-localization of TH and LIMK1 in different optic zones. Color scheme: green – LIMK1, red – TH, white – the areas of LIMK1 – TH co-localization. The scale bar size is 50 μm. See the Table for abbreviations and description of the optic zones.

The distribution of DAN clusters corresponded to that described in (Mao, Davis, 2009). PAM clusters were clearly observed near SMP, with processes extending towards the central part of the brain. The processes formed glomerular structures around the MB horizontal lobes (γ, β and β′L), probably being the synaptic endings innervating the correspond areas. The structure #3 was located above γL, the structures #5 and 8 – in the β′L area, the commissure #9 was seen in the central part of the brain. TH signal was relatively low in βL (see Suppl. Material 1). PPL1, PPM2 and other DAN clusters were observed around Cal, sending their processes to the different brain areas (Fig. 2, a).

LIMK1 was concentrated in the neuropil structures of the anterior part of the brain, such as SMP and AL. The LIMK1- positive granularity was observed inside SMP, between the β′L tips (#8) and around the ellipsoid body (EB) of CC (#10, 11). LIMK1 level was also high in thin tissue layers adjacent to neuropil and some neural tracts, such as #12 around the great commissure, #13 around wedge (W) and #15 near esophagus (ES), morphologically resembling glia (Hartenstein, 2011). LIMK1 signal was lower in cell bodies of the neurons surrounding AL (ALCB), probably being the cell bodies of the projection neurons, as well as in KC surrounding Cal. Here, LIMK1 was mainly concentrated in the cytoplasm, beyond the nuclei. LIMK1 level was significantly decreased in all the MB lobes and pedunculus (Ped), as well as in the CC structures, whereas in Cal and the protocerebral bridge (PB) it was relatively high (see Suppl. Material 1). LIMK1 and TH colocalization was observed in SMP, AL, Cal, the TH-positive cells and processes, and in glomerular densities, such as #3, 5 and 6 (see Fig. 2, b).

To check that the antibody specifically binds to LIMK1, the distribution of LIMK1 main product p-cofilin was assayed in CS. The pattern of p-cofilin distribution was generally similar to that for LIMK1 (Suppl. Material 2). The level of p-cofilin was low in MB (including Cal) and CC (mostly EB, as well as in the case of LIMK1). In contrast to LIMK1, p-cofilin was mainly concentrated in the cell nuclei in the peripheral area of the fly brain, such as Kenyon cells around Cal, as well as in PB, subesophageal ganglion (SEG), and cell bodies surrounding AL. p-Cofilin was also localized in diffuse layers within the brain structures, such as EB, probably formed by glia. The p- cofilin-enriched cells were found in SEG, forming structures with two glomerular branches (*), and around CC structures, fan-shaped body (FB) and EB (**).

Several GAL4 activators were used to initiate limk1 RNA interference. Both 6793 and 6794 strains specifically express the green fluorescent protein (GFP) under GAL4 promoter. In strain 6794, GAL4 was reported to be expressed in OL, thoracic ganglion, different nerves, and cortex glia (Sun et al., 1999; Okamoto, Nishimura, 2015). In 6794 > limk1-KD hybrid, GAL4-driven GFP expression was detected in glialike cells, surrounding the neuropil structures, such as AL, SMP, CC and its parts, as well as in the MB lobes, Ped and some KC (Suppl. Material 3, a). GFP level was higher in αL and βL compared to α′L, β′L and γL. The signal was lower in Cal and virtually absent in most neuropil structures, such as AL, SMP, CC and others. Thus, limk1 interference should occur in Cal and some glia cells, where the levels of both GAL4 and LIMK1 were relatively high. Similar distribution was observed in the control 6794 > limk1“+” strain (see Suppl. Material 3, b). In the strain 6793, GAL4 is expressed in cholinergic neurons (CHN), with GFP signal in OL, AL with the surrounding interneurons, the parts of CC, the great commissure (GC), Cal and the mechanosensory area of SEG (Salvaterra, Kitamoto, 2001). In the strains 6793 > limk1- KD and 6793 > limk1“+” (see Suppl. Material 3, c, d ), we observed a strong GFP signal in cell bodies surrounding SMP and AL, as well as in some KC, several neuropil structures (AL, α/βL, EB, FB), and GC. In all the studied strains, LIMK1 distribution character appeared to be similar to that in CS.

To check that GAL4 is active in 7009 and 30027 strains, we crossed them to strains expressing GFP under GAL4 promoter. In 7009 > 32186 hybrid, we observed a prominent GFP signal in cell clusters near SMP, morphologically similar to the TH-positive PAM clusters. Some cells might be SRN, but they constitute the minority of the observed neurons in this area (Albin et al., 2015; Kasture et al., 2018). The processes of PAM neurons extended to the horizontal MB lobes, including γL, and the densely innervated β′L tip (#5) connected by a commissure, and to a much lesser extent the βL tip (Suppl. Material 4, a). EB was surrounded by the GFP-positive processes extending from different parts of the brain. The GFPpositive DAN around Cal were also observed. Hence, GAL4 activator of 7009 should suppress limk1 inside DAN, including PAM neurons, which regulate memory storage in CCSP. The fruitless-positive neurons (FRN) are responsible for mating behavior. In 30027 > 32202 hybrid, we observed GFP in some KC, in the cell bodies located near SMP and AL, and glomerular structure, forming a ring-like structure around Ped (see Suppl. Material 4, b). Similar structures were described in (Yu et al., 2010). The distribution of LIMK1 in the hybrid strains with and without limk1 knockdown in the above neurons was similar to that in CS (Suppl. Material 5).

**Fig. 3.**
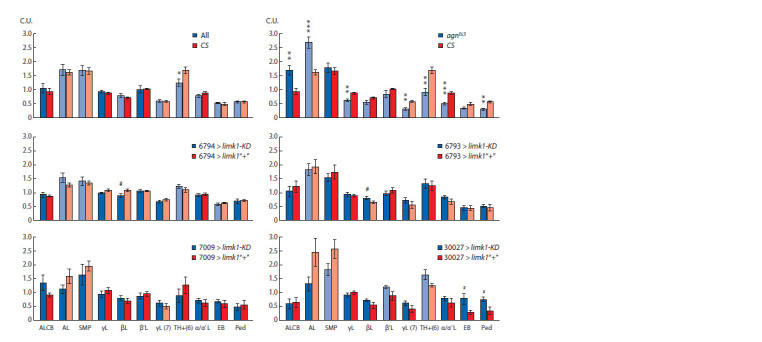
LIMK1 relative level in different brain structures The normalized levels are shown in conventional units (C.U.). All – the averaged brain of the all studied strains. Statistical differences (two-sided t-test): * from CS ( p < 0.05, 0.01, 0.001 for one, two and three asterisks, respectively); # from the control hybrid without limk1 interference; blue > cyan and red > pink color change – the difference from ALCB ( p < 0.05; blue and red – no difference). N = 47–61 (All), 5–6 (СS), 4–6 (agnts3), 7–8 (6794 > limk1-KD), 7–10 (6794 > limk1“+”), 5–6 (6793 > limk1-KD), 6–7 (6793 > limk1“+”), 3–5 (7009 > limk1-KD), 3–5 (7009 > limk1“+”), 4 (30027 > limk1-KD), 3–4 (30027 > limk1“+”). Standard error is shown.

The normalized intensity of LIMK1 signal was calculated for several brain structures (Fig. 3). The LIMK1 relative levels in specific structures were very similar for the CS brain and the average brain of all the strains. The biggest differences were observed for the TH-positive glomerular structure #6 (TH+(6)), which is possibly responsible for memory formation in CCSP. In the average brain, ALCB had the normalized LIMK1 level about 1. Compared to them, AL, SMP and TH+(6) structures had the higher LIMK1 level, whereas the MB lobes, EB and Ped had the lower LIMK1 level. In agn ts3, AL and ALCB had the higher LIMK1 level compared to CS, whereas most of the rest studies structures had lower LIMK1 level. This corresponds to more contrast LIMK1 staining in agn ts3 relative to CS (Suppl. Material 6). There were no prominent differences after limk1 knockout, except for several structures with minor changes. The interstrain differences might be local or beyond the resolution of the method.

3 h STM differs in hybrids with and without limk1 interference

**Fig. 4.**
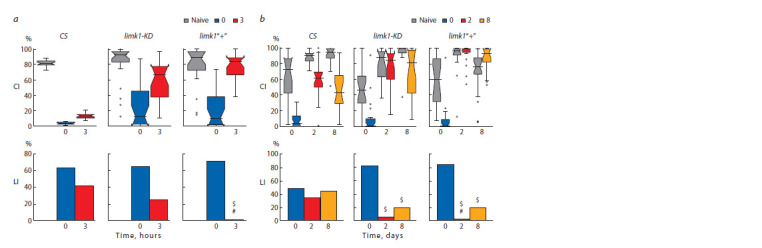
The memory abilities of the Drosophila strain with limk1 suppression by 6794 activator. a, STM. b, LTM. CI – courtship index; box plot and whisker chart, median is shown by the bold black line. LI – learning index. Statistical differences: # from CS, $ from LI (0 h/0 day) (two-sided randomization test; p < 0.05, n = 20).

3 h STM was estimated for limk1-KD (f ) > 6794 (m) and the control limk1“+” (f ) > 6794 (m) hybrids. In both cases, we observed the decrease of courtship index (CI) after learning, with its partial recovery after 3 h. The box plot height was minimal for CS and rather big for UAS × GAL4 hybrids, showing that the value of courtship suppression significantly varied for individual flies. All strains were capable to learn in CCSP, with learning index LI (0 h) immediately after training of about 60–70 % (Fig. 4, a). The CS LI was still high after 3 h, indicating STM preservation, in agreement with (Zhuravlev et al., 2022). The strain with limk1 interference also preserved STM: although its LI (3 h) was only about 20 %, it did not statistically differ from that for CS, as well as from LI (0 h). In the control strain, LI (3 h) decreased compared to LI (0 h) and did not differ from zero, indicating the impaired STM. Thus, while 3 h memory storage or retrieval was impaired in the control strain, limk1 interference seems to improve 3 h STM. At the same time, it did not affect the impaired 8 day LTM, with only minor positive effect on 2 day LTM (see Fig. 4, b).

Neuron type-specific limk1 interference differentially affects STM dynamics

**Fig. 5.**
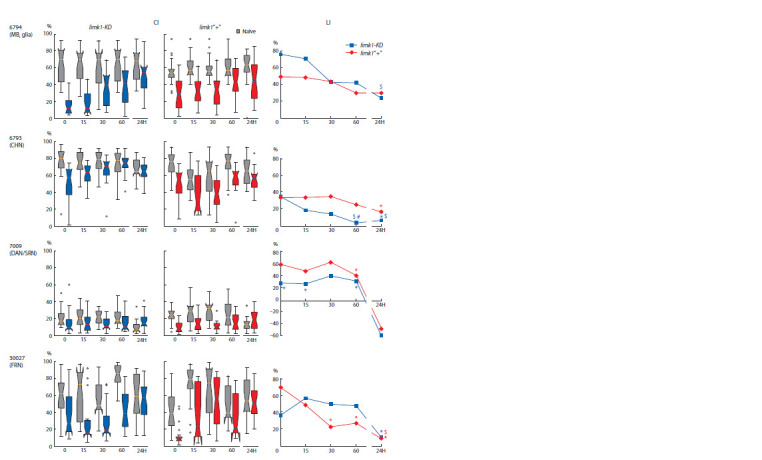
STM dynamics in Drosophila strains with neuron type-specific limk1 suppression CI – courtship index, LI – learning index. X axis: time (min; 24H – 24 hours). Statistical differences: # from CS, $ from LI (0 min), * no difference from zero (two-sided randomization test; p < 0.05, n = 20).

To investigate the dynamics of STM decay in different strains with limk1 interference, we performed LI analysis immediately and 24 h after learning (Fig. 5). To exclude the possible effect of eye color on learning and memory abilities, we applied GAL4 (f ) > UAS (m) crossing scheme, where both the strain with limk1 knockdown and the control strain had the same wild-type eye color. For 6794 activator (MB and glia), the control strain showed nearly the same LI within 24 h, whereas the strain with limk1 interference demonstrated a steeper forgetting curve. Hence, 6794 > limk1-KD showed high LI after learning, but seemed to increase the speed of memory forgetting on the interval 0–30 min. limk1 knockdown in CHN (6793) was associated with significant decrease of LI within 60 min after training.

For DAN and SRN activator (7009), both the strain with limk1 interference and the control strain showed nearly the same dynamics of STM decay, except for 30–60 min period. limk1 interference was associated with a dramatic defect on learning: LI did not differ from zero. LI (24 h) was negative in both hybrids, possibly being the effect of sensitization: males did not suppress the courtship activity but courted more actively some time after training. For FRN activator (30027), the effect of limk1 knockdown was the opposite to that of MB/glia and CHN activators: limk1 interference decreased the speed of forgetting, and LI (30 min) did not differ from zero. Thus, the effect of limk1 interference on STM dynamics appeared to depend on the neuronal type.

LIMK1 interference in CHN and FRN neurons differentially affects courtship song parameters

**Fig. 6.**
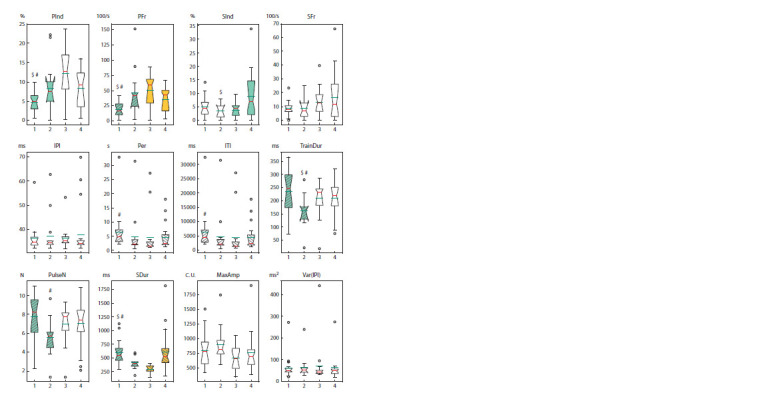
Courtship song parameters in Drosophila hybrids with neuron type-specific limk1 interference Hybrid numbers: 1. 6793 > limk1-KD; 2. 6793 > limk1“+” (CHN); 3. 30027 > limk1-KD; 4. 30027 > limk1“+” (FRN). Statistical differences: hybrids with limk1 interference vs control – the pairs with difference are shown by color (two-sided randomization test, p < 0.05) or hatching (two-sided Mann–Whitney test, p <0.05); hybrids with CHN driver vs hybrids with FRN driver: $ two-sided randomization test; # two-sided Mann–Whitney test. For hybrids 1, 2, 3, 4, respectively: n = 20, 15, 17, 22 for pulse song parameters; n = 18, 13, 15, 20 for sine song parameters. Median is shown by the red line, the mean value is shown by the green line.

Finally, we studied the influence of limk1 interference on the male courtship song parameters. The hybrids with CHN and FRN drivers were studied (Fig. 6). There were no interstrain differences in interpulse interval (IPI), the species- and population- specific parameter (Ritchie et al., 1994), and IPI variance (Var(IPI)), the marker of neurodegenerative processes (Savvateeva-Popova et al., 2003). limk1 interference in CHN (6793) decreased the pulse song index and frequency (PInd, PFr), increasing the mean period (Per), intertrain interval (ITI), train duration (TrainDur), sine song duration (Sdur) and train pulse number (PulseN). On the contrary, in the strain with FRN activator (30027), limk1 interference resulted in PFr increase, as well as Per, ITI, SInd and SDur decrease. limk1 knockdown by two different activators had the opposite effects on PInd, PFr, Per, ITI and SDur, leveling the initial differences between SInd, TrainDur and PulseN. Thus, limk1 interference in CHN seemed to decrease the rate of switching from the singing mode to silence mode and back, resulting in longer trains and ITI, while limk1 interference in FRN neurons generally had the opposite effect.

## Discussion

In mammals, LIMK1- and cofilin-dependent actin remodeling is widely involved in regulation of synaptic processes, such as exocytosis, receptor trafficking and remodeling of dendritic spines. These processes underlay long-term potentiation (LTP), long-term depression (LTD) and different forms of memory. LIMK1 also affects gene expression through CREB and LTM formation. Deregulation of LIMK1-dependent actin remodeling is involved in multiple pathologies, such as Alzheimer’s and Parkinson’s diseases, Williams syndrome, schizophrenia, and autism (Ben Zablah et al., 2021).

In mature neurons, actin is enriched in both pre- and postsynaptic structures, such as dendritic spines, regulated by Rho signaling pathway. The action of LIMK1 on actin polymerization and memory processes is rather complex, being dependent on the mode of LIMK1 regulation (transient or long-term overexpression) and cofilin level. While the active cofilin destabilizes fibrillar actin, in high concentrations it increases actin polymerization and nucleates actin filaments in dendritic spines during long-term potentiation (reviewed in Cuberos et al., 2015). Thus, it is hard to predict the integral effect of LIMK1 and cofilin activity on memory processes. It was crucial to check the behavioral effects of Drosophila limk1 suppression in specific types of neurons.

Using paraffin section staining, both LIMK1 and its product p-cofilin were shown to be homogeneously distributed in the brain neuropil, with maximum level in CC (Lopatina et al., 2007). Our results of the confocal microscopy analysis gave a different picture of LIMK1 distribution, quite similar for all the studied strains. We have shown a specific LIMK1 decrease in MB, which is responsible for associative learning, as well as in CC, involved in higher movement control (Strauss, Heisenberg, 1993). This puts under question the role of LIMK1 in the aforementioned processes. However, LIMK1 was present in the cell bodies and processes of DAN, which interact with MB and CC (Mao, Davis, 2009) and regulate memory and forgetting. The observed p-cofilin distribution resembled this for LIMK1: its level was low in the MB lobes and CC. The low level of p-cofilin in the MB lobes had been previously shown (Abe et al., 2014). In contrast to LIMK1, p-cofilin level was low in Cal formed by PN and KC terminals and high in cell nuclei. The latter corresponds to its functioning in the cell, as cofilin phosphorylation is necessary for its translocation into the nucleus (Abe et al., 1993).

The effectiveness of limk1 suppression at the RNA level was confirmed using Act-GAL4 activator in the whole Drosophila body. GAL4 was also active in specific brain areas of the corresponding strains. However, we failed to quantitatively check the changes of limk1 expression in Drosophila UAS × GAL4 hybrids with neuronal-specific GAL4 expression. The decrease in LIMK1 level might be local or too small. limk1 interference might also induce the compensatory activation of LIMK1 translation.

To study limk1 knockdown effects on memory, we used CCSP modification applied by (Kamyshev et al., 1999): training was performed with the mated female. In this case, courtship learning results from the rise of sensitivity to the antiaphrodisiac cis-vaccenyl acetate (cVA) due to unsuccessful courtship. cVA is not required for learning, being necessary for memory performance. aSP13 DAN, which innervate the fru-positive tip of γL, are necessary and sufficient for courtship learning (Keleman et al., 2012). 24 h memory consolidation requires the prolonged aSP13 stimulation and Orb2 dimerization in some γ neurons (Krüttner et al., 2015). α/β neurons are involved in LTM processes (Redt-Clouet et al., 2012; Jones et al., 2018). Hence, other DAN innervating α/βL of MB, including PAM and PPL1 cells (Aso et al., 2014a), may also be involved in LTM.

The behavioral differences were observed after limk1 interference, e. g., the restoration of 3 h STM for limk1-KD > 6794 strain. GAL4 drivers themselves affected memory abilities, which were generally decreased compared to CS. The drivers also significantly affected the forgetting curves. Thus, we studied the effects of limk1 interference relative to a control strain with the same GAL4 driver. We applied two different crossing schemes – UAS (f) > 6794 (m) (for 3 h STM and LTM analysis) and reverse (in the other behavioral experiments). In the first case, the control UAS > limk1“+” hybrid had bright eyes due to v[1] recessive allele, in contrast to UAS > limk1-KD hybrid with the wild type dark red eyes. The observed 3 h STM differences are unlikely to be associated with the differences in eyes pigmentation, as v[1] flies showed a normal 3 h STM and 2 day LTM in CCSP (Nikitina et al., 2021), while both forms of memory were impaired in the control strain. However, memory retention depends on parent affect, with some paternal epigenetic factors affecting STM strength (Medvedeva et al., 2021). For 6794 > limk1-KD strain, we did not see STM difference from the control strain, though learning ability slightly increased after limk1 knockdown (see Fig. 5). Thus, when studying LIMK1 effects on learning and memory, it is necessary to consider the crossing direction.

Acetylcholine is the major excitatory neurotransmitter in Drosophila. Among CNH are: PN forming synapses on KC of MB (Yasuyama et al., 2002), the MB intrinsic neurons that are responsible for olfactory memory, expressing ChAT and VAChT (Barnstedt et al., 2016), and the α/β core neurons required for LTM consolidation (Yi et al., 2013). In the hybrids with 6793 driver (GAL4 expressed in CHN), GFP level was specifically high in α/βL compared to the other MB lobes. Here, limk1 interference resulted in faster STM forgetting. This contradicts the cofilin role in active forgetting shown in OAVL, where cofilin was proposed to be regulated by LIMK1 (Shuai, Zhong, 2010). The involvement of LIMK1 and cofilin in forgetting may occur locally, within specific neuronal populations or synaptic terminals. At the same time, LIMK1 may be crucial for memory storage and retrieval in CCSP. The glutamatergic MBON M6 neurons serve for STM output: aSP13 DAN prolongs potentiation of γL – M6 synapses (Zhao et al., 2018). Some cholinergic MBON appeared to regulate the Drosophila visual appetitive memory (Aso et al., 2014b). As the extrinsic MB cells responsible for CCSP memory were similar to those used for appetitive memory (Montague, Baker, 2016), the decrease in 60 min STM might occur due to limk1 suppression in some of these neurons.

The hybrids with 7009 driver (DAN and SRN) showed generally low CI values and negative LI values 24 h after learning. Males of these strains had pale pink eyes because of defects of eyes pigmentation, due to non-complete w[1118] rescue. w[1118] males demonstrated low courtship activity and success, presumably due to some defects of sexual development and maturation (Xiao et al., 2017). However, the control 7009 > limk1“+” strain had normal LI up to 60 min after training. limk1 knockdown by 7009 driver was associated with dramatic defects of learning and memory: LI just after training did not statistically differ from zero. Thus, LIMK1- dependent signaling in DAN and SRN seems to be important for learning and memory in CCSP.

limk1 knockdown by 30027 driver (FRN) decreased the forgetting rate in the time interval 30–60 min. This corresponds to the role of actin-remodeling pathway in forgetting in OAVL paradigm (Shuai et al., 2010). LI of the control strain did not differ from zero starting from 30 min after learning, while limk1 knockdown increased it. In males, FRN are responsible for courtship behavior. There are ~1500 FRN in the Drosophila brain, including sensory organs, lateral horn, lateral protocerebrum, SMP arch and motor control centers. Together they provide multisensory integration to regulate the male courtship process (Yu et al., 2010; Liu et al., 2019). Some CHN and DAN are also Fru-positive, such as ~300 γL neurons and aSP13 DAN located in SMP, which regulates courtship learning and memory. The activity of fru gene was reported to decrease upon LTM formation in CCSP (Winbush et al., 2012). Hence, suppression of some FRN activity may be associated with memory prolongation and consolidation.

In addition to memory processes, limk1 interference affected some parameters of the male courtship song. As well as for courtship memory, we observed the opposite functional effects of limk1 knockdown in FRN and CHN. FRN of the P1 class initiate Drosophila courtship behavior and trigger courtship song. pIP10 neurons possibly convey the P1 signal to thoracic dPR1 and vPR6 neurons, proposed to be the parts of a central pattern generator (CPG), which defines the time and shape of the pulse song. vPR6 possibly encode IPI (von Philipsborn et al., 2011). Pulse and sine CPG either contain FRN or interact with them. As sine and pulse song normally do not overlap, the mutually inhibitory mechanisms must exist, switching between quasilinear and relaxation modes of oscillation for sine and pulse song, respectively. Some descending interneurons may control the type of the song, while the others trigger singing or terminate the song (Clyne, Miesenböck, 2008).

Indeed, we observed the opposite changes of PInd/PFr and SInd/SDur upon limk1 interference in CHN and FRN, moving the balance toward the sine and pulse song, respectively. The increase in PFr after limk1 knockdown in FRN might indicate the negative role of LIMK1-dependent signaling on activity of the pulse CPG or the upstream brain centers, which switch them from active to silent mode. СС is important for control of stability of pacemakers, which regulate the rhythmic structure of courtship song. PB destruction leads to sound signal distortions, FB and EB destruction additionally decreases sine and pulse trains (Popov et al., 2004). CC includes a large number of neuronal types, such as CHN, DAN, SRN, and others. CHN are present in FB, EB, No and PB (Kahsai, Winther, 2011), similarly to what we observed in our research. Hence, they probably play some role in regulation of male singing. The opposite effects of limk1 interference in CHN and FRN may indicate a specific role of LIMK1 in courtship controlling network, whereas the other parts of the brain possibly have a total antagonistic effects on its activity. Alternatively, CHN and FRN fru neurons may differ in some aspects of regulation of LIMK1-dependent signaling pathway.

## Conclusion

In summary, we have shown that effects of limk1 interference in Drosophila male courtship memory and song depend on both the neuronal type and specific behavioral parameter. limk1 interference in CHN and FRN had generally opposite effects, whereas its suppression in DAN and SRN impaired the flies’ ability to learn. Using activator strains with a narrower pattern of GAL4 expression would help to better localize the brain structures, where LIMK1 regulates memory and forgetting in CCSP. Among such putative structures are γL and aSP13 DAN innervating γ5 area, as well as other DAN participating in memory formation, consolidation and retrieval. Studying the behavioral consequences of limk1 overexpression in different brain areas will complement the estimates of the effects of its suppression. The above investigation should also focus on LIMK1 partner proteins, such as cofilin in its active and phosphorylated form.

## Conflict of interest

The authors declare no conflict of interest.
